# A Controlled Investigation of Optimal Internal Medicine Ward Team Structure at a Teaching Hospital

**DOI:** 10.1371/journal.pone.0035576

**Published:** 2012-04-19

**Authors:** Brad Spellberg, Roger J. Lewis, Darryl Sue, Bahman Chavoshan, Janine Vintch, Mark Munekata, Caroline Kim, Charles Lanks, Mallory D. Witt, William Stringer, Darrell Harrington

**Affiliations:** 1 Division of General Internal Medicine, Los Angeles Biomedical Research Institute at Harbor-University of California Los Angeles (UCLA) Medical Center, Torrance, California, United States of America; 2 David Geffen School of Medicine, University of California Los Angeles (UCLA), Los Angeles, California, United States of America; 3 Department of Emergency Medicine, Los Angeles Biomedical Research Institute at Harbor-University of California Los Angeles (UCLA) Medical Center, Torrance, California, United States of America; 4 Division of Pulmonary and Critical Care Medicine, Los Angeles Biomedical Research Institute at Harbor-University of California Los Angeles (UCLA) Medical Center, Torrance, California, United States of America; 5 Department of Medicine, Los Angeles Biomedical Research Institute at Harbor-University of California Los Angeles (UCLA) Medical Center, Torrance, California, United States of America; 6 Division of HIV Medicine, Los Angeles Biomedical Research Institute at Harbor-University of California Los Angeles (UCLA) Medical Center, Torrance, California, United States of America; Yale University School of Medicine, United States of America

## Abstract

**Background:**

The optimal structure of an internal medicine ward team at a teaching hospital is unknown. We hypothesized that increasing the ratio of attendings to housestaff would result in an enhanced perceived educational experience for residents.

**Methods:**

Harbor-UCLA Medical Center (HUMC) is a tertiary care, public hospital in Los Angeles County. Standard ward teams at HUMC, with a housestaff∶attending ratio of 5∶1, were split by adding one attending and then dividing the teams into two experimental teams containing ratios of 3∶1 and 2∶1. Web-based Likert satisfaction surveys were completed by housestaff and attending physicians on the experimental and control teams at the end of their rotations, and objective healthcare outcomes (e.g., length of stay, hospital readmission, mortality) were compared.

**Results:**

Nine hundred and ninety patients were admitted to the standard control teams and 184 were admitted to the experimental teams (81 to the one-intern team and 103 to the two-intern team). Patients admitted to the experimental and control teams had similar age and disease severity. Residents and attending physicians consistently indicated that the quality of the educational experience, time spent teaching, time devoted to patient care, and quality of life were superior on the experimental teams. Objective healthcare outcomes did not differ between experimental and control teams.

**Conclusions:**

Altering internal medicine ward team structure to reduce the ratio of housestaff to attending physicians improved the perceived educational experience without altering objective healthcare outcomes.

## Introduction

Over the past 15 years, the impact of internal medicine ward attending physician type (e.g., hospitalist vs. non-hospitalist) on health care and educational outcomes, and resident satisfaction with in-patient rotations has been evaluated [1], [2], [3], [4], [5], [6], [7], [8], [9], [10], [11], [12], [13], [14], [15], [16], [17]. However, one critical element that has been studied incompletely to date is the structure of the medical ward team at teaching hospitals (i.e. number of attending physicians, residents, and interns), and the impact of the ward team structure on the educational experience and objective performance measures.

It was hypothesized that ward team structures with a larger ratio of attending physicians to housestaff (i.e., residents and interns), and smaller overall team sizes would enhance the perceived educational experience as measured by resident and attending satisfaction with ward rotations, and possibly result in improved objective markers of quality of patient care.

## Methods

### Ward Teams and Admit Schedules

Harbor-UCLA Medical Center (HUMC) is a 400 bed academic, public teaching hospital serving a largely indigent patient population in urban areas of Los Angeles County. Five concurrently operating teaching internal medicine ward teams admit patients at HUMC. During the period of study (spanning academic years 2009–2011), each standard team was comprised of one attending physician, two residents (usually a PGY-2 and a PGY-3), and three interns. One attending physician was added to one of the five teams during each block period of study, enabling it to be converted into two smaller, experimental teams (Table 1), one of which had one attending, one resident, and one intern, and one of which had one attending, one resident, and two interns. All housestaff and attending physicians that participated in the experimental teams had previously staffed standard control teams. The standard three-intern teams admitted up to 15 new patients on each afternoon/evening call, and up to six new patients on each morning call. The two-intern and one-intern, experimental teams respectively admitted up to eight and five patients on afternoon/evening call and four and two patients on morning call, respectively. The emergency department and clinics were not notified when the experimental teams were implemented, and thus admitted patients to the teams by standard rotation without knowledge of whether patients were being admitted to experimental or standard teams. Patients admitted to the internal medicine inpatient ward teams but discharged from the emergency department or from clinics before reaching a ward bed were not included in the analysis. In addition, patients admitted to non-medicine services and then transferred to an internal medicine ward team after admission also were not included.

**Table 1:** Structure, Admissions, and Average Census of 3 Versions of Ward Teams Compared in the Current Study.

Team Structures				
	Attending	Resident	Intern	Attending ∶ Housestaff Ratio
Standard 3 Intern Team	1	2	3	1 ∶ 5
Experimental Team #1	1	1	2	1 ∶ 3
Experimental Team #2	1	1	1	1 ∶ 2

Admissions and Average Census Per Ward Teams Compared in the Current Study				
	New Admissions Per PM Call*	New Admissions Per AM Call*	Average Daily Census*	Maximum Daily Census (per ACGME intern cap)*
Standard 3 Intern Team	15	6	15	30
Experimental Team #1	8	4	10	20
Experimental Team #2	5	2	5	10

* For the attending physician.

### Data Gathering and Analysis

Web-based five-point Likert satisfaction surveys were developed by the Internal Medicine training program leadership (i.e., Program Director and Associate Program Directors) in collaboration with the Director of Graduate Medical Education and the Chairman of the Department of Medicine. The survey metrics focused on goals set internally to improve the educational experience of the rotation. These goals included: to improve the perceived educational value of the rotation by housestaff and attending physicians, to increase bedside teaching by attendings, to improve the quality of the intern-resident interactions, and to increase time for bedside rounding and patient care while maintaining or decreasing time spent in the hospital overall. The survey was sent electronically, via the internet, to all residents and attending physicians on both the experimental and control teams, the day their rotations ended. The surveys asked respondents to compare their current experience to prior experiences with standard teams.

We hypothesized closer supervision enabled by the smaller team structure could lead to improved care, more rapid decision-making, and more appropriate discharges. Thus, as objective measures of medical care and quality, in-patient mortality, length of stay in hospital, and same-hospital readmission rates were compared between patients cared for on the experimental and control teams. Data for these objective measures were obtained by electronic query of the hospital information system.

To ensure that patient admissions to the experimental and control teams were similar, patient age, gender, emergency department triage scores (which incorporate disease severity), and case mix index (CMI) were compared between the experimental and control ward teams. CMI was defined as the average relative weight of the Medicare Severity-Diagnostic Related Group (MS-DRG) assigned to the patients by hospital coders post-discharge on the experimental or control teams. All of these comparative data elements, including the CMI, was obtained by electronic query of the hospital information system after all of the patients charts were coded. The study was approved by the John F. Wolff, MD, Human Subjects Committee Institutional Review Board of the Los Angeles Biomedical Research Institute at Harbor-UCLA Medical Center, and was conducted according to the principles expressed in the Declaration of Helsinki.

### Statistical Analysis

We sought to capture data from 200 admissions to the combined experimental teams during the interventional periods, and from 800 admissions to the standard teams. These sample sizes were chosen to yield 80% power to detect (two-tailed α = 0.05) a 0.9 day reduction in median length of hospital stay (based on a median length of stay for patients on the internal medicine ward service at Harbor-UCLA of 4 days). Length of stay was chosen as the basis for power calculation because it had the most robust baseline data available of the planned quality of care measures.

Continuous and interval data were summarized using median and interquartile ranges (IQRs) and compared using the nonparametric Wilcoxon rank sum test. Nominal variables were summarized using proportions and percentages and compared using the chi square or Fisher's exact test. The primary comparisons were made between the combined results from both experimental teams and control teams.

## Results

### Admissions to the Experimental and Standard Teams

The ward team experiment was conducted on three separate occasions: February 1–28, 2010, September 16–30, 2010, and January 9–February 12, 2011. For scheduling reasons, the middle experiment was run for only a two week period instead of a full month. Thus, the experiment was conducted for a total of 12 weeks (2.5 month-long blocks). Data from all three periods were combined for analysis and interpretation. During these periods, 184 patients were admitted to the experimental internal medicine services (81 to the 1 intern teams and 103 to the 2 intern teams) and 990 were admitted to the standard teams (Table 2). Median age, triage acuity in the emergency department, and case mix index for patients on the experimental and the standard teams were similar (Table 3).

**Table 2:** Number of Admissions for Experimental and Standard Ward Teams.

Period	# (%) Admissions to Experimental Team 1 Intern Team ∶ 2 Intern Team	# Admissions to Standard Teams
01 Feb to 28 Feb 2010	81 (16%)	441 (84%)
	44 (8%) ∶ 37 (7%)	
16 Sep to 30 Sep 2010	31 (13%)	200 (87%)
	12 (5%) ∶ 19 (8%)	
9 Jan to 12 Feb 2011	72 (17%)	349 (83%)
	25 (6%) ∶ 47 (11%)	
**Total**	**184 (16%)**	**990 (84%)**
	**81 (7%) ∶ 103 (9%)**	

**Table 3:** Comparison of Patients on Experimental vs. Standard Teams.

	Experimental Teams	Standard Teams	P
Median (IQ range) Age (yrs)	Combined: 54 (47, 63)	54 (44, 63)	0.39
	1 Intern Teams: 54 (44, 61)		
	2 Intern Teams: 55 (49, 65)		
Median (IQ range) Emergency Room Triage Acuity (score 1–3)	Combined: 2 (2, 2)	2 (2, 2)	0.70
	1 Intern Teams: 2 (2, 2)		
	2 Intern Teams: 2 (2, 2)		
Rate (95% CI) of Step Down Unit Admissions	Combined: 31% (25–38%)	32% (30–35%)	0.74
	1 Intern Teams: 32% (22–42%)		
	2 Intern Teams: 29% (20–38%)		
Rate (95% CI) of ICU Admissions	Total: 8% (4–12%)	7% (6–9%)	0.60
	1 Intern Teams: 6% (0–11%)		
	2 Intern Teams: 10% (4–15%)		
Case Mix Index	0.97 (0.69, 1.46)	0.97 (0.72, 1.45)	0.87
	0.91 (0.68, 1.19)		
	1.03 (0.67, 1.47)		

* IQ = interquartile, CI = confidence interval.

### Resident and Attending Likert Satisfaction Scores

Residents and attending physicians had very similar impressions of the experimental ward teams (Tables 4 and 5). When comparing them to previous rotations on standard ward teams, both resident and attending physicians responded that the experimental ward teams afforded more time for attending teaching and for bedside teaching, and resulted in better resident-intern interactions, a better overall educational experience, a better quality of life, more time for patient care, and superior training for hospital-based medical practice (Tables 4 and 5). Resident physicians also indicated that they spent less time rounding with the attending on the experimental teams than they had previously on standard teams. However, the attending physicians on the experimental teams did not report spending less time rounding.

**Table 4:** Resident Likert Satisfaction Survey*.

	Experimental Teams	Standard Teams	P
Educational Value of Rotation, % improved or much improved	Total: 81% (13/16)	30% (12/40)	<0.001
	1 Intern Teams: 88% (7/8)		
	2 Intern Teams: 75% (6/8)		
Bedside Teaching by Attending, % more or much more	Total: 75% (12/16)	40% (16/40)	0.02
	1 Intern Teams: 75% (6/8)		
	2 Intern Teams: 75% (6/8)		
Quality of Resident-Intern Interactions, % improved or much improved	Total: 88% (14/16)	38% (15/40)	<0.001
	1 Intern Teams: 88% (7/8)		
	2 Intern Teams: 88% (7/8)		
Time for Attending Teaching, % more or much more	Total: 75% (12/16)	15% (6/40)	<0.001
	1 Intern Teams: 75% (6/8)		
	2 Intern Teams: 75% (6/8)		
Time for Patient Care, % more or much more	Total: 88% (14/16)	8% (3/40)	<0.001
	1 Intern Teams: 75% (6/8)		
	2 Intern Teams: 100% (8/8)		
Time Spent Rounding, %less or much less	Total: 63% (10/16)	13% (5/40)	<0.001
	1 Intern Teams: 63% (5/8)		
	2 Intern Teams: 63% (5/8)		
Quality of Life, %improved or much improved	Total: 50% (8/16)	8% (3/40)	<0.001
	1 Intern Teams: 63% (5/8)		
	2 Intern Teams: 25% (2/8)		
Preparation for Hospital-Based Medicine, %improved or much improved	Total: 81% (13/18)	38% (15/40)	<0.003
	1 Intern Teams: 88% (7/8)		
	2 Intern Teams: 75% (6/8)		

* 1 = much worse or less than previous rotations; 2 = worse or less than previous rotations; 3 = same as previous rotations; 4 = improved or more than previous rotations; 5 = much improved or much more than previous rotations.

**Table 5:** Attending Likert Satisfaction Survey*.

	Experimental Teams	Standard Teams	P
Educational Value of Rotation, % improved or much improved	Total: 100% (8/8)	11% (1/9)	<0.001
	1 Intern Teams: 100% (4/4)		
	2 Intern Teams: 100% (4/4)		
Bedside Teaching by Attending, % more or much more	Total: 100% (8/8)	11% (1/9)	<0.001
	1 Intern Teams: 100% (4/4)		
	2 Intern Teams: 100% (4/4)		
Quality of Resident-Intern Interactions, % improved or much improved	Total: 75% (6/8)	11% (1/9)	0.01
	1 Intern Teams: 75% (3/4)		
	2 Intern Teams: 75% (3/4)		
Time for Attending Teaching, % more or much more	Total: 88% (7/8)	0% (0/9)	<0.001
	1 Intern Teams: 100% (4/4)		
	2 Intern Teams: 75% (3/4)		
Time for Patient Care, % more or much more	Total: 100% (8/8)	0% (0/9)	<0.001
	1 Intern Teams: 100% (4/4)		
	2 Intern Teams: 100% (4/4)		
Time Spent Rounding, %less or much less	Total: 38% (3/8)	11% (1/9)	0.24
	1 Intern Teams: 50% (2/4)		
	2 Intern Teams: 25% (1/4)		
Quality of Life, %improved or much improved	Total: 88% (7/8)	0% (0/9)	<0.001
	1 Intern Teams: 100% (4/4)		
	2 Intern Teams: 75% (3/4)		
Preparation for Hospital-Based Medicine, %improved or much improved	Total: 100% (8/8)	0% (0/9)	<0.001
	1 Intern Teams: 100% (8/8)		
	2 Intern Teams: 100% (8/8)		

* 1 = much worse or less than previous rotations; 2 = worse or less than previous rotations; 3 = same as previous rotations; 4 = improved or more than previous rotations; 5 = much improved or much more than previous rotations.

### Experimental versus Standard Ward Team Objective Outcome Measures

The median length of stay (82 vs. 81 hours, p = 0.52), hospital charges ($22,172 vs. #22,172, p = 0.97), 15- (9% vs. 9%, p = 0.52) and 30-day (13% vs. 12%, p = 0.71) readmission rates, and mortality rates (1.6% vs. 2.7%, p = 0.16) were similar when comparing the combined experimental teams versus the standard ward teams, respectively. There were also no significant differences between 1 intern and 2 intern experimental ward teams.

## Discussion

This study strongly supports that ward team structure has a fundamental and direct impact on the ability of attending physicians to supervise and teach housestaff. Reducing the ratio of housestaff per attending on the ward team resulted in a superior perceived educational experience and substantially improved resident and attending satisfaction. No objective differences in healthcare outcomes were observed. Additional research is required to identify interventions to ward team structure that could result in improved objective healthcare outcomes.

Graduate medical education has become an important driver for improved quality and patient safety in the hospital. Accordingly, the Accreditation Council for Graduate Medical Education (ACGME) has placed increasing emphasis on training requirements which improve educational experience related to health care quality and patient safety. The 2008 Institute of Medicine (IOM) report on resident duty hours affirmed that limitations to resident work hours and improvement in resident supervision are key to promoting high-quality education and safe patient care [18]. In many programs, reducing resident work hours required transfer of some patient care activities to other providers. However, limited resources have forced many training programs to develop novel, restructured teams to meet both resident work hour and supervision requirements.

Compliance with these new work hour standards is measured both objectively and subjectively. While a variety of objective measures exist, subjective and thus perceived responses of trainees have become increasingly important. In fact, ACGME requires trainees to document their perceptions regarding compliance with work hours, supervision, quality of the training program, as well as, individual well-being at least annually via a web-based survey. Therefore, training programs must be cognizant of the potential impact of changing the training environment to meet accreditation standards on perceived satisfaction of trainee and faculty.

The marked superiority of satisfaction with the educational experience and quality of life by both residents and attending physicians in the current study make the experimental teams attractive for full implementation. Nevertheless, experimental teams would require additional resources to implement, since they require more attending physicians to supervise the same number of housestaff and staff the same number of patients. The recent change in resident work hour requirements for interns mandated by ACGME, which limit continuous in-hospital duty to no more than 16 consecutive hours, has stretched resources even further for internal medicine ward teams. Furthermore, decreasing housestaff hours requires additional housestaff to staff ward teams, or forces housestaff teams to care for fewer patients. In either case, more attending physicians would be required to achieve the smaller ratios of housestaff to attending physicians ratios on the experimental teams as described in the current study. Balancing available resources with the increasing demands placed on teaching services by ACGME work hour rules and the desire to optimize the perceived educational experience of housestaff is becoming increasingly challenging. If housestaff and attending physicians perceive that superior educational experiences are achieved by having smaller ward teams, but smaller ward teams become increasingly difficult to maintain due to the reduced work hours and increasing requirements for time spent out of the hospital, it is possible that changing ACGME work hour rules without additional resources may adversely impact educational experiences on inpatient internal medicine wards.

After we initiated our ward team experiment, McMahon et al. published a similar investigation comparing ward team structures on in-patient internal medicine teaching services [19]. In their intervention, team structures were compared with one experimental team having two attending physicians, two residents, and three interns (housestaff to attending ratio of 5∶2) vs. two control teams having one intern, one resident, and two interns (housestaff to attending ratio of 3∶1). Thus, the control teams studied by McMahon et al. were similar in size to the experimental teams in the current study. The current study resulted in a much larger reduction in patient load on objective outcomes than was analyzed in the study by McMahon et al. Nevertheless, our results were concordant with the previous study. Both demonstrated improvements in resident satisfaction and quality of educational experience with the experimental teams. In the current study attending physicians also felt that experimental teams resulted in superior educational experiences.

The primary limitations of the study are its single center design and comparison of overall mortality/length of stay rather than risk-adjusted. Although mortality and length of stay were not risk-adjusted, there was no difference in case mix index between experimental and control teams, suggesting that risk-adjustment would not modify the outcomes. Also, the survey instrument used was not validated prior to deployment, and was rather developed based on goals we had set internally to improve the educational experience of the rotation. Finally, we analyzed only same-hospital readmission rates, and cannot exclude the possibility that readmissions occurred at neighboring hospitals. However, the patients on the experimental and control teams were similar by key demographics, so there is no a prior reason to suspect an imbalance of patients more likely to be admitted to neighboring hospitals on the experimental vs. the control teams.

In summary, decreasing the ratio of housestaff to attending physicians and decreasing the patient census per attending resulted in a perceived enhanced educational experience with a better quality of life for residents and attending physicians. Institutions and training programs must understand the value and impact of proposed solutions when attempting to comply with evolving accreditation standards. Perception of the training environment by both the learner and teacher will likely remain an important determinant in academic medicine.
